# Inheritance of Susceptibility to Malignant Blood Disorders

**DOI:** 10.1038/s41598-019-38879-y

**Published:** 2019-02-21

**Authors:** Viggo Jønsson, Haneef Awan, Neil D. Jones, Tom B. Johannesen, Bjarni á Steig, Gudrid Andosdottir, Geir E. Tjønnfjord

**Affiliations:** 1 Department of Hematology, Oslo University Hospital, KG Jebsen Center for B cell malignancies P.O. box 4950, Nydalen, NO 0424 Oslo, Norway; 2 Institute of Clinical Medicine, University of Oslo, P.O. box 4950, Nydalen, NO 0424 Oslo, Norway; 3 Department of Research Computing, University of Oslo, P.O. box 1059, Blindern, NO 0316 Oslo, Norway; 4 Department of Computer Science, University of Copenhagen, Universitetsparken 5, Building B, DK 2100 Copenhagen, Denmark; 5 Norwegian Cancer Registry, Ullernchausseen 64, NO 0379 Oslo, Norway; 6 National Hospital of the Faroe Islands, Medical Department, J.C. Svabos gøta 1, FO 100 Torshavn, Faroe Islands; 7 Genetic Biobank of the Faroe Islands, J.C.Svaboe gøta 43, FO 100 Torshavn, Faroe Islands

## Abstract

Malignant blood disorders depend on heritable susceptibility genes and occur in familial aggregations. We suggest a model of transgenerational segregation of the susceptibility genes based on the study of malignant blood disorders in Norwegian and Danish families with unrelated parents, and in the inbred Faroese population with related parents. This model, consisting of parental genomic imprinting and mother-son microchimerism, can explain the male predominance in most of the diseases, the predominance of affected parent-offspring when parents are not related, and the different modes of segregation in males and females. The model displays a specific pattern in the distribution of affected relatives for each diagnosis, viz. a characteristic distribution in the pedigrees of family members with malignant blood disorder related to the proband. Three such patterns, each reflecting a specific transgenerational passage, were identified: (1) alterations in the number of affected relatives in paternal lines alone, e.g. in patterns for probands with multiple myeloma; (2) alterations in the number of affected relatives in both paternal and maternal lines for probands with chronic lymphocytic leukemia; and (3) no alterations in the numbers of male and female affected relatives in the parental lines, e.g. for probands with some types of malignant lymphoma.

## Introduction

Strong evidence supports the notion that leukemia, lymphoma, myeloma and other malignant blood disorders (MBD) in man constitute an entity of heritable diseases^1–7^, but the transgenerational transfer of the susceptibility to MBD is still largely unknown. Heritable susceptibility genes, so called risk genes, are necessary for the production of tumor in the form of a mutated blood cell monoclone^8,9^. A repertoire of susceptibility genes for each diagnosis comprises one or a few specific genes combined with a number of unspecific genes that differ from patient to patient with the same diagnosis. Genome-wide association studies clearly show this so-called “genomic landscape”, e.g. in the lymphoproliferative diseases (LPD) including chronic lymphocytic leukemia (CLL), acute lymphoblastic leukemia (ALL), Hodgkin’s lymphoma(HL), non-Hodgkin’s lymphomas (NHL) and multiple myeloma (MM); and in the myeloproliferative diseases (MPD), e.g. chronic myeloid leukemia (CML), acute myelogenous leukemia (AML), myelofibrosis (MF), polycythemia vera (PV), and essential thrombocytosis (ET)^10–17^. There are silent bystanders within this polygenic model of susceptibility (persons having congenic susceptibility without developing the pathognomonic mutation), and there is a range of aggressiveness (stage of disease) in patients with the same diagnosis, so that low-stage diseases with weak or no symptoms are easily overlooked and not included in the registration of hereditary, familial MBD. Genealogical studies of MBD in families^18,19^, and large-scale screenings from cancer registries^20–24^ confirm a wide variety of different diagnoses within LPD and MPD (pleiotropy), but show no clear signs of a Mendelian or other specific pattern in the transgenerational transfer. Until now, no satisfactory explanation of the predominance of males has been given, e.g. in CLL. Further open questions concern anticipation (increased severity of malignancy down through the generations and low age at onset of disease)^4^, and sib ship birth order effect (non-random in the ages of affected siblings)^25^, and a pronounced inconsistency with regard to co- and contravariation of diagnoses (degree of linking)^23,24^.

We now describe a novel model of the segregation of susceptibility to MBD. Based on new genealogical investigations of a large number of families with MBD, we are able to recognize distinct transgenerational pathways of the susceptibility genes and to identify the mechanisms likely involved in the inheritance of MBD.

## Material and Methods

Our study is based on a genealogical investigation of familial MBD in two groups. One group comprises 315 patients from the inbred population of the Faroe Islands. The other group comprises 301 patients from 112 families with MBD and unrelated parents from Norway and Denmark. In both groups, cross-checked pedigrees were used for study. For comparison, diagnosis frequencies were extracted from the National Cancer Registries in Norway and Denmark.

### The Faroe Islands

#### Patients and diagnoses

Based on data from the Diagnostic Registry in Torshavn (capital of the Faroe Islands), all 341 known cases of MBD from the Faroe Islands up to December 2011 were considered for study. In a five year period after 2011, it was ensured that all patients with delayed diagnoses were enrolled after proper cross-check. Three patients failed inclusion due to lost medical record or insufficient data for the verification of MBD, and 23 patients failed because their exact position in the family tree was uncertain due to extramatrimonial relationship and unauthorized adoption. They hardly represent a selective loss, the diagnoses were MM 4, AML 4, ALL 3, CLL 3, NHL NOS 3, ML 3, DLBCL 2, CML 1. (Names and abbreviations of diagnoses, cf. Table 1) Thus, 315 patients (LPD 219, MPD 87, others 9, Table 1) were included after cross-check, comprising a review of the medical record and the pathologist’s SNOMED registration^26^ related to clinical diagnosis, laboratory findings, radiological assessments, symptoms and course of disease. The diagnosis of each patient was reassessed according to the diagnostic criteria valid at the time of diagnosis. In malignant lymphomas, for example, the Faroese Diagnostic Registry includes during the observation period cases according to the American Registry of Pathology (1934), Rappaport- (1956, 1966), Lennert- (1967), Kiel- (1974), WHO- (1976) Working- (1982) and Real- (1994) classifications^8^. Therefore, in order to obtain a common denominator for the study group, all included patients were grouped according to the ICD-10 diagnostic system^27^. NOS-diagnoses (not otherwise specified) have been used in 45 cases: 25 cases where the cross-check could not definitely confirm the subtype of NHL (NHL NOS, ICD-10 C85.9), in 15 cases in which a clear distinction between myelodysplasia (MDS) and other types of MPD was not ascertained (ML NOS, ICD-10 C92.9), and in five cases of lymphoid leukemia in elderly desolate patients who died without further diagnostic procedure (Leukemia NOS, ICD-10 C91.3–91.5).

**Table 1:** Diagnoses.

Diagnoses	Familial Diagnoses Observed							Cancer Registries
ICD-10 code numbers	The Faroe Islands			Norway and Denmark				Norway and Denmark
Lymphoproliferative Disorders	Pc			Pc				All cases recorded
	Total	(males, females)	%	Total	(males, females)	%		% (mean)
Hodgkin’s lymphoma HL C81	19	(12, 7)	8.7	11	(8, 3)	4.0		6
Follicular lymphoma FL C82	14	(9, 5)	6.4	19	(13, 6)	6.9		12
Mantle cell lymphoma MCL C82.7	0			4	(4, 0)	1.4		<1
Diffuse Non-Hodgkin’s lymphoma DLBCL C83.3	34	(21, 13)	15.6	16	(9,7)	5.8		25
Peripheral T-cell lymphoma TNHL C84	6	(2, 4)	2.7	2	(2, 0)	0.7		2
Monocytoid B-cell lymphoma MONOC C85.7	0			4	(1, 3)	1.4		2
Non-Hodgkin lymphoma NOS NHL NOS C85.9	25	(14, 11)	11.4	9	(4, 5)	3.3		6
Waldenström’s disease WA C88	2	(1, 1)	0.9	8	(5, 3)	2.9		3
Multiple myeloma MM C90	50	(31, 19)	22.8	10	(6, 4)	3.6		14
Acute lymphoblastic leukemia ALL C91.0	16	(10, 6)	7.3	4	(2, 2)	1.4		4
Chronic lymphocytic leukemia CLL C91.1	50	(29, 21)	22.8	181	(98, 83)	65.6		22
Prolymphocytic leukemia PLL C91.3	2	(1, 1)	0.9	2	(0, 2)	0.7		1
Hairy cell leukemia HCL C91.4	1	(1, 0)	0.5	1	(0, 1)	0.4		1
Large granular T-cell leukemia LGTCL C91.7	0			3	(2, 1)	1.1		<1
Monoclonal gammopathy MGUS D47.2	0			2	(1, 1)	0.7		2
Lymphoproliferative disease LPD total	219	(131, 88)	100	276	(155, 121)	99.9		100
Male/Female ratio		1.5			1.3			
Age at onset of disease Years (median)	61	(58, 64)		67	(65, 69)			
Birth order effect		NO			Patrilineal CLL, only			
**Myeloproliferative Disorders**								
Acute myeloblastic leukaemia AML C92.0 and C92.2-9	44	(28, 16)	50.6	9	(6, 3)		37.6	70
Chronic myeloid leukemia CML C92.1	14	(9, 5)	16.1	3	(1, 2)		12.5	15
Polycythemia vera PV D45	4	(2, 2)	4.6	6	(4, 2)		25.0	5
Myelodysplasia MDS D46	2	(0, 2)	2.3	2	(1, 1)		8.3	5
Myelofibrosis MF D47.1	8	(4, 4)	9.2	1	(0, 1)		4.2	5
Essential thrombocytosis ET D47.3	0			3	(1, 2)		12.5	<1
Myeloid leukemia, uncertain ML NOS C92.9	15	(9, 6)	17.2	0				<1
Myeloproliferative disease MDP total	87	(52, 35)	100	24	(13, 11)		99.9	100
Male/Female ratio		1.5			1.2			
Age at onset of disease Years (median)	54	(49, 60)		63	(59, 68)			
Birth order effect		NO			NO			
**Other Malignant Disorders**								
Leukemia, uncertain L NOS C95.9	7	(2, 5)		1	(1, 0)			
Malignant histiocytosis MH C96.1	2	(0, 2)		0				

Comments: Pc, Proband crude, number of patients observed.

NOS, not otherwise specified.

To assure anonymity, the patients were numbered for study and no name, initials or birthplace were used so that no recognition from the outside was possible. For each patient, data on gender, age at entry in the study, year of birth, age and year of diagnosis, aliveness or age and year at death, and the birth rank order of the patient with the number and gender of older and younger sibs were available. There were no twins among the patients.

#### Families and pedigrees

The National Civil Registry in Torshavn provided the pedigrees showing the position of patients with MBD and their kinship with unaffected family members over 5 to 6 generations in one big family. Progeny Software was used for the processing and storage of the pedigrees^28^. The 315 included patients were designated Proband crude (Pc).

#### Population and sampling history

The genetic founders are the nearly two thousand descendants of the original Vikings who survived the Black Death around 1350 and reproduced themselves in about 20 generations to the approximately 50,000 inhabitants of today^29^. Before 1900 foreign settlers, mainly European traders, crafts, Danish priests and administrative staff, constituted a minority with minimal effect on the Faroese genome. One hundred years ago, the influx of foreign people, mainly from other Scandinavian countries, was only about 10 persons per 5 years^29^. The population increased from about 15,600 people in 1900 to about 49,700 in 2011, and the mean lifespan increased from about 45 years in 1900 to 82 years in 2011^29^. In this inbred population, fishermen and sailors from abroad and later British soldiers during WW II who found a bride on the Faroe Islands most often brought the females home, so the Faroese genome remained largely unaffected^30^. The Coefficient of Inbreeding has never been calculated^30^.

The Faroese parity is unique. The mean number of live births per female was 7.4 in 1900 and 2.7 in 2011, some of the highest figures in Europe^31^. The age of the mother at first delivery was nearly the same as in Norway^32^ and in Denmark^33^. The late fetal death rate per 1000 live births was 11.0 on the Faroe Islands compared with 5.0 in Norway and 5.6 in Denmark when investigated in the late 1990s^31^. Estimated from data on first born singletons, the mean Faroese live born birth weight is significantly higher (3610 g plus/minus standard deviation 609 g) than that of Norway (3520 g plus/minus 574 g) and that of Denmark (3411 g plus/minus 585 g), the live born weight increasing with mother’s age and parity^31^. These data on parity and birth weight are used in the discussion of growth factors (cf. Discussion).

The oldest patient included was a female with CLL, born 1884. Data from the Cancer Registries in Norway and Denmark was used for comparison with the familial cases. Within the observation period, the diagnostic intensity in the main islands appears to be nearly the same as in Denmark and Norway, but lower elsewhere on the Faroe Islands, especially in the beginning of the period and on small, remote islands far out in the ocean with few or no general practitioners. Apart from the last few decades there has been no tradition for a systematic report to the National Danish Cancer Registry in Copenhagen, and no Faroese cancer statistic on incidence and prevalence is available.

All methods were carried out in accordance with relevant guidelines and regulations concerning Cancer Registry Data and all protocols were approved by relevant licensing authorities (cf. Acknowledgements). Further, all patients were included with informed consent (cf. Informed consent). Participants under the age of 18 years were included with informed consent from a parent or a legal guardian.

### Norway and Denmark

#### Patients and diagnoses

Our joint database on familial MBD has 301 patients (LPD 276, MPD 24, other 1) up to December 2011 (Table 1). These families were observed for new cases of MBD in a five year period after 2011. All diagnoses were cross-checked with reports from the pathology departments, medical records of the hospitals, and registrations in the national cancer registries in Norway and Denmark. The diagnoses were sorted according to the ICD-10 nomenclature^27^, in the same way as described for the Faroese material. All patients belonged to the genuine Scandinavian population of Norway and Denmark. There were no twins and no consanguinity. The first known cases of CLL, a 51-year-old man born in Norway in 1872 with CLL since 1923, and a 54-year-old female from Denmark, born 1864 with CLL in 1918 were included.

#### Families, population and sampling history

The 301 patients were extracted from 112 families in our database with two or more cases of MBD and unrelated parents. Several thousands of MBD patients have been screened for familial disease during the past two decades or more. From an overall assessment, our database is extensive in the sense that a large and representative number of patients have been available for the present study.

The parity in Norway and Denmark is nearly the same. The mean number of live births per female was 4.2 in 1900 and 1.7 in 2011^32,33^. The mean late fetal death rate per 1000 live births was 5.3 in the late 1900s^31^. However, parity comprises children born alive, stillborn, and abortions where especially the early spontaneous abortions, experienced as a late, strong menstruation, are practically impossible to enumerate. Taken together, about 15% of all pregnancies end with a miscarriage in Scandinavia^32,33^, but that figure too, is uncertain when estimating parity related to the mother in a mother-son microchimeristic scenario, because such elusive factors as the number of male partners and expected changes in male fertility during the observation period exert their influence.

All methods were carried out in accordance with rules and regulations concerning Cancer Registry Data (cf. Acknowledgements) and all patients were included with informed consent (cf. Information consent). Participants under the age of 18 years were included with informed consent from a parent or a legal guardian.

### Statistics

#### Diseases

The MBD-patients observed in the Faroese family and in MBD-families from Norway and Denmark were compared with “a normal material” based on data from the whole population National Cancer Registries in Oslo^34^ and Copenhagen^35^, and from the LYFO-Registry^36^ (Table 1). The diagnoses observed were expressed in per cent of the total LPD and total MPD, respectively, and the percentages (frequencies) were used for comparison of the findings in the Faroese family with the families from Norway and Denmark, and for comparison with the normal material. Significance was accepted for P < 0.05. Sex- and age-adjusted incidences and prevalences would have been ideal, but these parameters are not available in the Faroese material and cannot reliably be constructed retrospectively as described (cf. Material and Methods, the Faroe Islands, Population and sampling history). Age at onset of disease in the two groups of familial MBD was described and compared by means of two-sided Mann-Whitney’s test.

#### Genealogy

**Figure 1:**
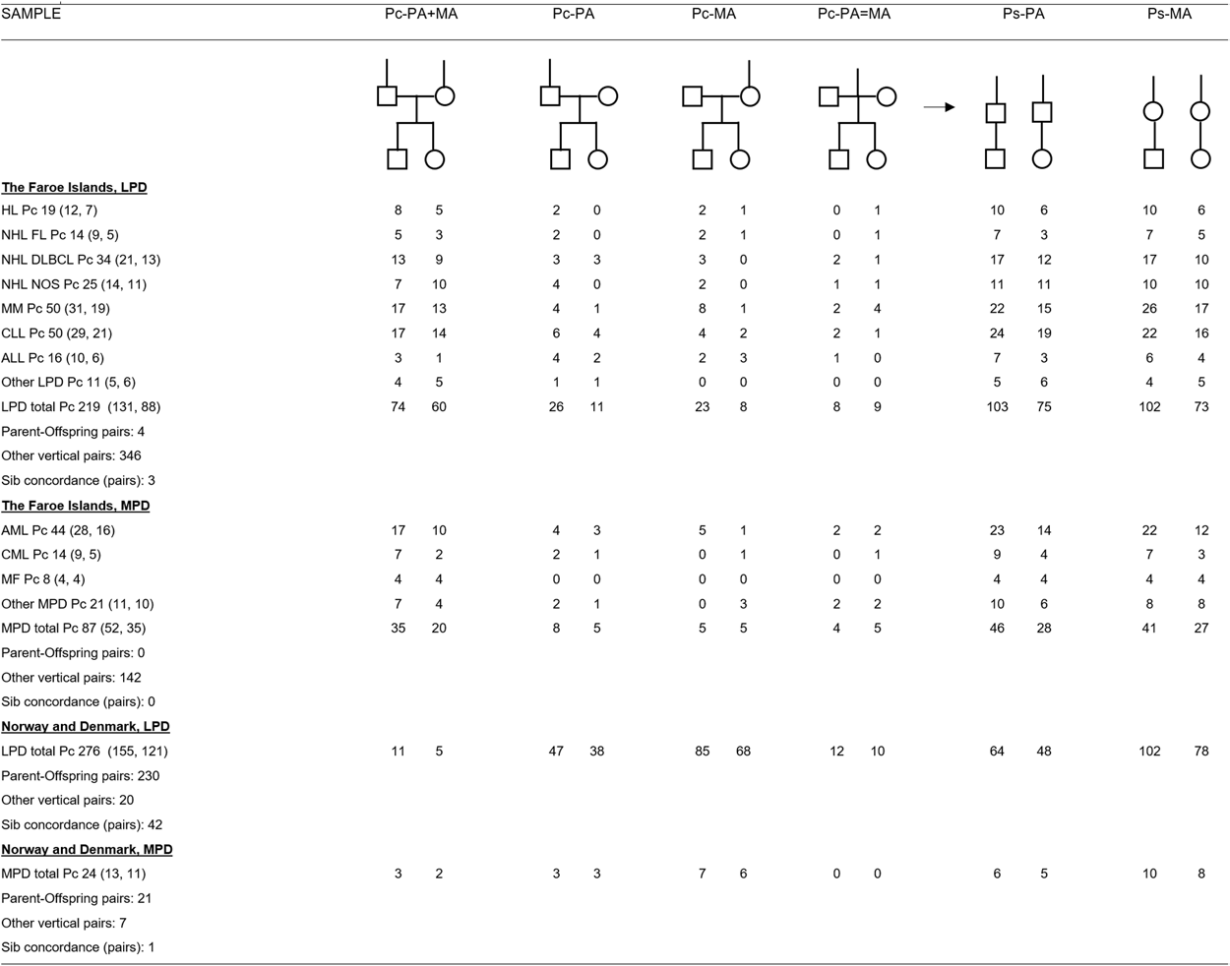
Pc and Ps probands and their parental affiliation. Pc, (Proband crude, number of patients observed: total (males, females)). Ps, (Proband standard, number of patients according to monoparental affiliation). Arrow shows the conversion of Pc to Ps. PA and MA, patrilineal and matrilineal; PA = MA, mixed or uncertain parental affiliation; Other vertical pairs than Parent-Offspring, for example, Proband-Uncle, Proband-Aunt pairs. A given Pc can act in several combinations of pairs at the same time, for example in sib concordance and concomitantly in a Parent-Offspring pair. Therefore, the number of pairs is not half the number of patients. Abbreviations, diagnoses (cf. Table 1): LPD, lymphoproliferative disorders; Other LPD, T-NHL, Burkitt-NHL, WA, PLL and HCL; MPD, myeloproliferative disorders; Other MPD, PV, MDS, ML NOS.

Each patient with familial MBD (Table 1) was appointed Proband crude (Pc) and the number, diagnoses and position in the pedigree of his or her affected relatives (AR) were recorded (Fig. 1, Table 2).

**Table 2:** Affected Relatives and Proband Pc and Ps, The Faroe Islands.

Affected Relatives (mean)/Pc					Affected Relatives Total (males, females) in Ps groups					
	m/m	f/m	m/f	f/f		Psm-PA	Psm-MA	Psf-PA	Psf-MA	Totals
LPD/HL	5.7	4.4	9.7	7.6	OBS	65(37, 28)	25(13, 12)	13(6, 7)	18(12, 6)	121(68, 53)
					EXP	30(17, 13)	30(17, 13)	30(17, 13)	30(17, 13)	121(68, 53)
					**OBS/EXP (P)**	**3(3, 3)**	**0(0, 0)**	**−2(−2, 0)**	**−1(0, 0)**	
LPD/NHL FL	4.9	2.2	8.8	4.0	OBS	13(12, 1)	19(12, 7)	7(2, 5)	25(18, 7)	64(44, 20)
					EXP	16(11, 5)	16(11, 5)	16(11, 5)	16(11, 5)	64(44, 20)
					**OBS/EXP (P)**	**0(0, 0)**	**0(0, 0)**	**−1(−2, 0)**	**0(0, 0)**	
LPD/NHL DLBCL	4.7	3.9	7.6	6.3	OBS	59(32, 27)	42(23, 19)	39(24, 15)	41(20, 21)	181(99, 82)
					EXP	45(25, 20)	45(25, 20)	45(25, 20)	45(25, 20)	181(99, 82)
					**OBS/EXP (P)**	**1(0, 0)**	**0(0, 0)**	**0(0, 0)**	**0(0, 0)**	
LPD/NHL NOS	5.9	3.7	7.5	4.7	OBS	28(19, 9)	37(22, 15)	39(23, 16)	30(18, 12)	134(82, 52)
					EXP	34(21, 13)	34(21, 13)	34(21, 13)	34(21, 13)	134(82, 52)
					**OBS/EXP (P)**	**0(0, 0)**	**0(0, 0)**	**0(0, 0)**	**0(0, 0)**	
LPD/MM	5.8	4.3	9.5	7.0	OBS	102(63, 39)	88(46, 42)	46(23, 23)	77(48, 29)	313(180, 133)
					EXP	78(45, 33)	78(45, 33)	78(45, 33)	78(45, 33)	313(180, 133)
					**OBS/EXP (P)**	**2(2, 0)**	**0(0, 0)**	**−3(−3, 0)**	**0(0, 0)**	
LPD/CLL	5.0	3.2	7.0	4.5	OBS	82(53, 29)	77(51, 26)	43(19, 24)	38(23, 15)	240(146, 94)
					EXP	60(37, 23)	60(37, 23)	60(37, 23)	60(37, 23)	240(146, 94)
					**OBS/EXP (P)**	**2(2, 0)**	**1(1, 0)**	**−1(−2, 0)**	**−2(−1, 0)**	
MM/CLL	(Small sample size)				OBS	21(14, 7)	17(10, 7)	13(9, 4)	4(4, 0)	55(37, 18)
					EXP	14(9, 5)	14(9, 5)	14(9, 5)	14(9, 5)	55(37, 18)
CLL/CLL	(Small sample size)				OBS	15(10, 5)	14(7, 7)	12(9, 3)	10(4, 6)	51(30, 21)
					EXP	13(8, 5)	13(8, 5)	13(8, 5)	13(8, 5)	51(30, 21)
LPD/ALL	3.5	1.6	5.8	2.7	OBS	19(15, 4)	10(6, 4)	7(3, 4)	15(11, 4)	51(35, 16)
					EXP	13(9, 4)	13(9, 4)	13(9, 4)	13(9, 4)	51(35, 16)
					**OBS/EXP (P)**	**0(0, 0)**	**0(0, 0)**	**0(−1, 0)**	**0(0, 0)**	
LPD/other LPD	(Small sample size)				OBS	11(8, 3)	4(2, 2)	25(9, 16)	12(5, 7)	52(24, 28)
					EXP	13(6, 7)	13(6, 7)	13(6, 7)	13(6, 7)	52(24, 28)
LPD/LPD	5.2	3.6	7.7	5.4	OBS	382(239, 143)	299(175, 124)	219(111, 108)	256(153, 103)	1156(678, 478)
					EXP	289(170, 119)	289(170, 119)	289(170, 119)	289(170, 119)	1156(678, 478)
					**OBS/EXP (P)**	**3(3, 1)**	**0(0, 0)**	**−3(−3, 0)**	**0(0, 0)**	
MPD/LPD	4.0	2.7	5.9	4.0	OBS	85(60, 25)	64(40, 24)	25(7, 18)	63(34, 29)	237(141, 96)
					EXP	59(34, 24)	59(34, 24)	59(34, 24)	59(34, 24)	237(141, 96)
					**OBS/EXP (P)**	**3(3, 0)**	**0(0, 0)**	**−3(−3, 0)**	**0(0, 0)**	
LPD/MPD	3.2	2.4	4.7	3.6	OBS	91(54, 37)	79(44, 35)	50(21, 29)	71(45, 26)	291(164, 127)
					EXP	73(41, 32)	73(41, 32)	73(41, 32)	73(41, 32)	291(164, 127)
					**OBS/EXP (P)**	**1(1, 0)**	**0(0, 0)**	**−2(−2, 0)**	**0(0, 0)**	
MPD/MPD	2.8	1.7	4.2	2.5	OBS	95(66, 29)	66(45, 21)	28(10, 18)	47(27, 20)	236(148, 88)
					EXP	59(37, 22)	59(37, 22)	59(37, 22)	59(37, 22)	236(148, 88)
					**OBS/EXP (P)**	**3(3, 0)**	**0(0, 0)**	**−3(−3, 0)**	**0(0, 0)**	
MPD/AML	2.5	1.7	4.4	3.0	OBS	41(27, 14)	32(19, 13)	17(7, 10)	28(17, 11)	118(70, 48)
					EXP	30(18, 12)	30(18, 12)	30(18, 12)	30(18, 12)	118(70, 48)
					**OBS/EXP (P)**	**1(1, 0)**	**0(0, 0)**	**−1(−2, 0)**	**0(0, 0)**	
MPD/CML	3.4	2.4	6.2	4.4	OBS	21(15, 6)	12(9, 3)	8(1, 7)	12(6, 6)	53(31, 22)
					EXP	13(8, 5)	13(8, 5)	13(8, 5)	13(8, 5)	53(31, 22)
					**OBS/EXP (P)**	**1(1, 0)**	**0(0, 0)**	**0(−1, 0)**	**0(0, 0)**	
MPD/other MPD	3.1	1.1	3.4	1.2	OBS	26(19, 7)	16(13, 3)	2(0, 2)	2(2, 0)	46(34, 12)
					EXP	12(9, 3)	12(9, 3)	12(9, 3)	12(9, 3)	46(34, 12)
					**OBS/EXP (P)**	**3(3, 1)**	**0(0, 0)**	**−2(−2, 0)**	**−2(−1, 0)**	

Comments: Abbreviations, cf. Tables 1 and 2. m, males; f, females.

OBS/EXP (P): observed versus expected (P, chi-squared test).

Zero: observed not different from expected (P > 0.05).

Positive values, observed higher than expected.

Negative values, observed lower than expected.

At the following levels: P < 0.05 score 1, P < 0.01 score 2 and P < 0.001 score 3.

The pedigrees of all Faroese Pc were manually formatted into one united pedigree to describe the inbred family in which the AR were registered only once with ties of kinship in paternal (PA) and/or maternal (MA) lines (Fig. 1). The AR associated a given Pc in the united Faroese pedigree were recorded with up to five healthy family members between Pc and AR in vertical (parental) lines and/or in other vertical, oblique lines (uncle, aunt). The AR included were allocated to one of the following groups (1) AR in both patrilineal and matrilineal lines (PA + MA), (2) in PA only (PA), (3) in MA only (MA), or (4) entirely mixed or unknown (PA = MA), which means that ancestors to the proband of father’s and mother’s lines are mixed due to inbreeding to such an extent that their parental affiliation could not be determined (Fig. 1). In the same way, Fig. 1 also shows the parental affiliation added from the smaller 112 families with unrelated parents from Norway and Denmark.

A given AR was registered only once per Pc in either PA or MA, the one chosen being the one closest to the proband. The united pedigree with the total overview of the large, inbred Faroese family made it feasible to rule out double entry of a given AR to the same Pc. However, a given patient in the pedigree could act as AR to several Pc, especially in parts of the pedigree with many patients, viz. a high density of Pc and many shared AR. This explains why the total number of observed AR (Table 2) can be significantly higher than the total number of Pc (Table 1), both in LPD and in MPD. Data from the smaller Norwegian and Danish MBD-families shows the same tendency although the number of shared AR per Pc is much lower (301 Pc from 112 MBD-families) than in the Faroese material.

In order to expose a common pattern in the transgenerational transfer of MBD-susceptibility, the united Faroese pedigree was used as a master for inferential visual pattern recognition^37,38^ to identify the mechanism behind the distribution of AR and Pc. We considered all relevant mechanisms we could think of (the Mendelian modalities, genomic imprinting, mother-son and mother-daughter microchimerism, gene linkage etc.). There are four different groups of Pc-AR with different parental affiliation (Fig. 1). In order to estimate paternal and maternal inheritances separately as part of the pattern recognition, a uniform, monoparental assessment of AR was required. Proband standard (Ps) denotes such a monoparental affiliation of AR to a given Pc. In contrast to Pc, Ps counts only one parental affiliation with its ARs in either patrilineal (Ps-PA) or in matrilineal (Ps-MA) lines. The following guidelines were used to convert Pc to Ps: Group (1), Pc having ARs in both father’s and mother’s lines at the same time gave rise to twice the number of Ps, one for PA and one for MA. In Group (2) and (3), the number of Pc was equal to Ps. Data from Group (4) was allocated to either Ps-PA or to Ps-MA based on an estimation of which parental line that brought Ps and the majority of AR closest together. In other words the lowest number of healthy family members between Ps and AR compared in the father’s and mother’s line in the pedigree. Some 21 Faroese Pc (12 males and 9 females) had a very peripheral position in the pedigree so that no affected predecessors could be seen. These cases of unknown parental affiliation were shared equally between Ps-PA and Ps-MA. The numbers of observed ARs are reported in Table 2, denoted OBS.

The number of OBS (Table 2) was compared with the expected number of AR (total, males, females), denoted EXP (Table 2). EXP (the null-hypothesis) was based on an equal distribution of the AR among the four groups: Psm-PA, Psm-MA, Psf-PA and Psf-MA, viz. 25% of OBS for each of the four groups.

The birth order effect, i.e., the rank in the sib ship by age of affected sibs, was estimated for every Pc by means of the Haldane Smith test^25^, in which the sum of rank numbers in the test sample (6A) was compared with the theoretical value and expressed as the 95% confidence interval (CI 95%). The Wilcoxon signed-ranks test was used for control, comparing the sum of positive (older) and negative (younger) rank numbers of affected sibs in the sib ship with zero representing the median age of all healthy and affected sibs in the sib ship.

To estimate anticipation, the number of patients in each diagnosis in the familial material (“observed”) was compared with the National Cancer Registry findings (“expected”) by chi-squared test.

Co- and contra variation in the Faroese material was estimated by means of a systematic comparison of AR per Pc, observed versus expected (chi-squared test, significance P < 0.05).

Statistical analysis was done with programming language R version 3.3 (www.r-3-3project.org).

### Informed Consent

The patients were informed about the purpose of the study, that it was free to decline, of course, and that the investigation was approved by the Scientific Ethical Committee. Furthermore, that data would remain confidential and unrecognizable outside the study, and that data could be crosschecked with the National Cancer Registry and with the Civil Person Registry if necessary. Each patient confirmed participation by completing a questionnaire, approved by the National Ethical Committees.

## Results

### Diagnoses

The myeloproliferative disorders are more frequent in the Faroe material (28% of all MBD) than in the families from Norway and Denmark (8%). (Table 1) AML is predominant but with a frequency lower than expected when comparing with the percentage of AML reported by the whole-population Norwegian and Danish Cancer Registries (P < 0.05). In contrast, CML has nearly the same frequency in the families and in the cancer registries.

Regarding the lymphoproliferative pleiotropic diversity, CLL is increased in the Norwegian and Danish families compared with the percentage of CLL reported by the cancer registries (P < 0.01). In contrast, the percentage of MM is increased on the Faroe Islands (P < 0.01) where MM is as common as CLL. A reduction of familial DLBCL compared with the occurrence of DLBCL in the cancer registries is confirmed in both the Norwegian and Danish families (P < 0.01) and in the Faroese family (P < 0.05). A near halving of FL is seen in families with MBD (P < 0.05), even when the uncertainty related to NHL NOS is taken into account (50% united NHL, ICD-10 C82–C85, reported by the cancer registries compared with 19.6% of the Norwegian and Danish families, and 36.1% of the Faroese families).

HL is increased in familial MBD, compared with the figures from the cancer registries, reporting 6% HL out of all LPD and 12% HL out of all malignant lymphomas (HL plus NHL) in Norway and Denmark. The Norwegian and Danish families showed 16.9% HL among all malignant lymphomas, and the Faroese family showed 19.4% (P < 0.01).

The percentage of ALL reported by the cancer registries is not significantly different from the percentage in the Norwegian and Danish families (P > 0.05) while in the Faroes material, the proportion of ALL is nearly twice as high (P < 0.01).

The Faroese material has the most pronounced signs of malignancy and anticipation, for example the highest frequency of ALL and the lowest frequency of some indolent lymphomas, e.g. FL, and the lowest age at onset of disease, LPD (P < 0.05), MPD (P < 0.01). Male patients had in general a lower mean age at onset of disease than female patients in both the Faroes and in the Norwegian and Danish material, LPD (P < 0.05), MPD (P < 0.01). The male/female ratio is higher on the Faroe Islands (P < 0.05).

A significant birth order effect is only observed in the Norwegian and Danish CLL-families related to patrilineal inheritance where affected sons appeared late in the sib ship (P < 0.05).

### Probands and parental affiliation

Each patient referred to in Table 1 was appointed as Proband crude (Pc). (Fig. 1) The positions in the pedigree of a patient’s affected relatives (AR) were counted as described in section “Material and Methods, Statistics, Genealogy”: PA + MA (AR in both parental lines; PA (paternal only); MA (maternal only); and PA = MA (mixed or uncertain). Analysis of Ps (Pc formatted into monoparental lines) in the inbred Faroese family showed that the number of Ps-PA and Ps-MA is not significantly different in LPD (P > 0.05) and MPD (P > 0.05), and that Ps-males are predominant compared with Ps-females in both in PA and MA (P < 0.01). Parent-offspring affected pairs are rare compared with oblique affected pairs between Ps and uncle, aunt, denoted “other vertical pairs”, LPD 98%, MPD 100% (P < 0.001).

In contrast, the Norwegian and Danish families with unrelated parents shows that Ps-MA is larger than Ps-PA (LPD: P < 0.05; MPD: same tendency with small sample sizes). In both PA and MA, parent- offspring pairs are predominant (P < 0.01).

### Probands and affected relatives

All LPD and MPD in the Faroese material have an entirely stereotypic pattern where Pc-females have more AR than Pc-males (P < 0.001), and the AR/Pc ratio m/m is larger than f/m (P < 0.001) and m/f is larger than f/f (P < 0.001) (Table 2 to the left).

(Table 2) In AR/Ps (Table 2 to the right), OBS is the number of AR counted in the pedigree for Pc and transformed into AR/Ps. EXP is the excepted ratio AR/Ps. The expected number of AR has been calculated as one fourth of the total observed AR equally distributed among the four Ps-groups Psm-PA, Psm-MA, Psf-PA and Psf-MA (cf. Material and Methods, Statistics, Genealogy). OBS and EXP were compared using chi-squared test.

AR/Ps displays three distinct patterns of distribution (PD). Each PD has a characteristic configuration of the probands and their affected relatives, males and females, related to the parental affiliations. (1) The most common PD, seen in LPD/MM, LPD/LPD, MPD/LPD, LDP/MPD, MPD/MPD, MPD/AML and MPD/CML, has in PA a higher number of AR-males related to Ps males and fewer AR-males related to Ps females than expected while in MA, the numbers of AR-males and AR-females are as expected. (2) A PD for Ps-CLL with increased male-AR to Ps-males in both PA and MA combined with decreased male-AR related to Ps-females in both PA and MA. This tendency was also seen in MM/CLL, CLL/CLL and LPD/other LPD, even though consisting of only small sample sizes. Finally, (3) a PD for NHL and ALL, where the numbers of AR-males and AR-females were generally not different from what was expected in PA and MA.

In contrast to the Faroese AR, belonging to one big family, there are 112 Norwegian and Danish families with a total of 301 MBD-patients, so that the mean number of MBD-patients per family is only 2.7, and the mean number of AR per Pc, (301-112)/112), is 1.7, too small for a reliable comparison with the huge numbers of AR in the Faroese material. However, taking data from Table 2 into account, it can be concluded that the parental affiliation of AR to Norwegian and Danish Ps is stronger in MA than in PA in LPD and MPD (P < 0.01), compared with an equal affiliation to PA and MA in the Faroese material (P > 0.05).

### Co- and contravariations

All diagnoses have been systematically screened for co- and contra variation, comparing observed data versus expected. In HL, for example, Pc is 19 (12, 7), total (males, females), and the total of Pc-LPD is 219 (131, 88) (Table 1). The number of AR to HL is 121 (68, 53) (Table 2). Thus, if AR were equally distributed, the expected number of total AR would be 19 × 121/219 = 10.5 compared with the observed 19 (chi-squared test = 4.90). Observed versus expected males and females were compared in the same way. Significant results from the Faroe Islands:

PcMM-contravariation AR: CLL.

PcMM-covariation AR: ALL, CML and ML NOS.

PcDLBCL-covariation AR: DLBCL and MM.

PcDLBCL-contravariation AR: CLL.

PcNHL NOS-covariation AR: HL and DLBCL.

PcAML-covariation AR: AML, MM and DLBCL.

PcAML- contravariation AR: CLL and ML NOS.

PcCLL-co- and contravariation AR: none.

Covariation of the same diagnosis in Pc and AR (clustering): HL, DLBCL, MM, AML.

## Discussion

**Figure 2:**
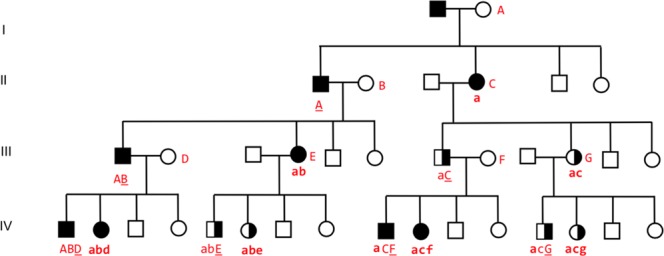
Maternal imprinting and mother male-feto microchimeristic genomic change. Proposed model of the segregation of susceptibility. Maternally imprinted susceptibility genes: Black square (affected male) or black circle (affected female); combined black and white (carrier); white (normal person) in four generations (I–IV). Mother-son microchimeristic changes of susceptibility genes: red capital “A to G” (strong expression of susceptibility); red small letters “a to g” (weak expression of susceptibility). Capital at the same line as the mother (acquired microchimeristic genomic change in the mother); red capital underlined (male inducer of microchimerism). Anticipation can be seen as an increased microchimeristic genomic load (red letters) down through the generations. Manifest disease depends on a certain load of susceptibility genes, for example black signature (imprinted genes) and at least two red letters (microchimeristic genomic changes). Paternal (PA) lines: Manifest male disease accumulates e.g. in the father-son line to the left in the figure from IIIAB to IVABD and further on in the PA males in the next generations with highly expressed microchimeristic genomic changes (red capitals only). And in the PA lines of sons of carrier fathers, e.g. IVaCF and further down in the next generations, all with partial expression of the microchimeristic genomic changes (mixed red capital and red small letters). Thus, the male probands in PA lines have a greater number of male affected relatives than female probands, because the female probands create carriers that break up the continuous lines of affected males and thereby reduce the number of available affected relative males per female proband. Maternal (MA) lines: An equal number of carrier males and females are produced from affected females. Transgenerational lines of female carriers are produced in MA lines, e.g. to the right in the figure from IIIacG to IVacg and further on in the subsequent generations of MA.

**Figure 3:**
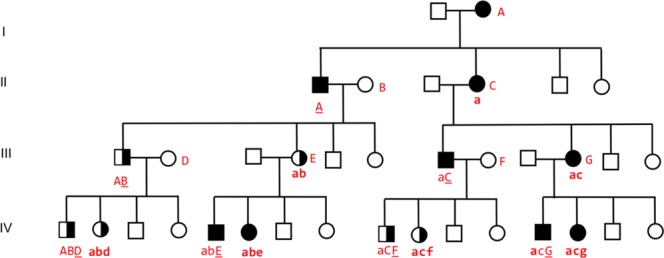
Paternal imprinting and mother male-feto microchimeristic genomic change. Proposed model of the segregation of susceptibility. Paternal imprinting, otherwise the same signature as for Fig. 1. Paternal lines (PA): An equal number of carrier males and females are produced from carrier males under the formation of a continuous male carrier line (to the very left in Fig. 2). Maternal lines (MA): Accumulation of manifest female disease, e.g. in the mother-daughter line to the right in the figure from generation IIaC to IIIacG to IVacg and further on to the females in MA lines in the next generations. And in the MA lines of females of carrier mothers, e.g. IVabe and down with the females in MA lines in the subsequent generations, all with predominantly low expression of microchimeristic genomic changes (mainly small red letters). All affected males are produced in MA lines, and the affected males have in general a stronger expression of microchimeristic genomic changes than the affected females.

New cumulative data from families with multiple cases blood disorders (MBD) reveal three patterns of the distribution (PD) of the patients in the family (Table 2). Assuming that a systematic description of the mutual positions of the patients in the pedigree to some extent reflects the segregation of the genetic susceptibility to MBD, pattern recognition^37,38^ exposes parental genomic imprinting^39^ together with mother-offspring microchimerism^40–44^ as the likely mechanisms involved in the transgenerational segregation of the susceptibility genes. Figures 2 and 3 were used to describe the interaction of these mechanisms in the conversion of the static PD into the dynamic operative algorithms (pathways).

Pathway 1 contains a higher number of male affected relatives (AR) related to male-Ps (proband standard) and fewer AR-males related to female-Ps in paternal (PA) lines than expected, and no significant changes in maternal (MA) lines (cf. Results, Proband and Affected Relatives, Table 2). Maternal imprinting combined with mother-son microchimerism can explain an increasing number of affected males with highly expressed susceptibility (Fig. 2) and seems a plausible explanation of the surplus of males in LPD/MM, LPD/LPD, MP D/LPD, LDP/MPD, MPD/MPD, MPD/AML and MPD/CML (Table 2). These PA-lines receive in addition affected males (e.g. IVaCF in Fig. 2) from father carriers (e.g. IIIaC) where the sons will proceed into the next generations as PA-lines with affected males in every generation. In contrast, affected females are produced only in every other generation on one line of descent with low expressive microchimeristic genomic changes, with offspring along the MA-lines that break up the continuous lines of AR-males available per female proband. Thus, affected females in PA-lines will have fewer affected males available, “spread of the AR in the pedigree”, than the affected males (Table 2).

Concomitant paternal imprinting and mother-son microchimerism (Fig. 3) of susceptibility genes from the genomic landscape of the diagnosis can explain the presence of affected mother-offspring pairs in a functionally balanced interaction between PD 1 and PD 2. A near equal number of affected males and females are produced. The affected males have a relatively weak expression of microchimeristic genomic changes (mixed red capital and red small letters) and no males have a strong expression (red capital only) like that seen in maternal imprinting. In other words, the microchimeristic genomic changes in males and females are more alike in MA-lines than in PA-lines. This can explain the non-significant difference when comparing the distribution of observed (OBS) males versus expected (EXP) males in MA-lines of PD 1 and pathway 1.

Pathway 2, related to CLL, has increased male-AR to male-Ps in both PA and MA-lines combined with decreased male-AR related to female-Ps in both PA and MA-lines (cf. Results, Proband and Affected Relatives, Table 2). Concomitant maternal imprinting and mother-son microchimerism is assumed to exert its action as described above related to PD1 and pathway 1. In CLL, however, especially in families with unrelated parents, affected mother-son pairs are the most frequent transgenerational combination (Fig. 1), indicating a stronger affinity of mother’s susceptibility to sons than to daughters. That concerns the paternal imprinting and its mother-son microchimerism in such a way that affected males become predominant (e.g. in the combinations from IIaC to IIIaC, and from IIIacG to IVacG, Fig. 3) explaining the PD 2 in MA of pathway 2. Here, the production of affected males by paternal imprinting, restricted to MA-lines (Fig. 3), is higher (more intensive) than expected, viz. higher than an equal distribution of AR, expressed by assigning one fourth of the total AR to each of the four Ps-groups Psm-PA, Psm-MA, Psf-PA and Psf-MA. That’s exactly what is seen in the distribution of male-AR to CLL female-Ps in MA-lines (e.g. LPD/CLL, Table 2).

Pathway 3, related to NHL and ALL (Table 2), exhibits an expected number of affected males and females in both PA and MA-lines, which is easily explained as a merge of Figs 2 and 3 where affected males and females have nearly the same microchimeristic genomic expression (A = a, B = b, C = c etc.) without predominance of affected males.

Figures 2 and 3 depict a situation with stronger microchimeristic genomic charge in males (red capitals) than in females (red small letters) but otherwise, the pathways are assumed to act in concert depending upon the MBD-diagnoses related to the pleiotropic susceptibility genes present in the patient (“the genomic landscape”). Gradual deviations from the pathways, for example in LPD/HL (Table 2), are theoretically expected in advance due to degrees of gene expressivity (penetrance), variations in trigger-environmental epigenetic stimuli, co- and contravariation, and the number and range order by age of the sons in the sib ship. By means of the AR/Pc ratios as a kind of control (Table 2 to the left) it is easy to see that the ratios AR/Pc-females are generally higher than AR/Pc- males as expected when the number of affected females is lower than the number of affected males (Table 1). Ratios with AR males are generally higher than ratios with AR females as expected when the number of affected males is higher than the number of affected females (Table 1), especially in CLL^8,45–48^. The increased number of male CLL-patients has been a mystery for decades so far, there have been no evident hormonal or any other pathophysiological explanations.

The genetic mode of segregation in the three pathways emerged while trying to match our findings against all known Mendelian and non-Mendelian modalities. Parental genomic imprinting combined with mother-son microchimerism appeared as a comprehensive correct solution bringing about a strong match, especially when both maternal and paternal imprinting are combined. Maternal imprinting causes accumulation of susceptibility in males (Fig. 2 to the left) and the formation of female carrier lines (to the right). Paternal imprinting causes accumulation of susceptibility in females (Fig. 3 to the right) and the formation of male carrier lines (to the left). Pattern recognition suggests that one combine both mechanisms to explain the presence in the pedigrees of affected father-offspring lines (in Fig. 2) and affected mother-offspring lines (in Fig. 3).

The many small families with unrelated parents from Norway and Denmark with a mean of only about 3 patients per family would never deliver such a clear PD as the Faroese with 315 patients in one family. However, the families from Norway and Denmark deserve attention because they support the idea of the model (Figs 2 and 3) that the degree of tissue-type compatibility (microchimerism) is relevant. We got a better match between our data and mother-son microchimerism than mother-son added daughter microchimerism. That could well be in accordance with the fact that mother-son microchimerism is the strongest due to difference in gender (“the gender barrier”). Thus, affected mother-son pairs are the most frequent parent-offspring combination in families with unrelated parents (Fig. 1). A birth order effect late in patrilineal sib ships was only seen in male CLL with unrelated parents (Table 1) at that precise place where mother’s acquired and accumulated tolerance to HLA of the father and of the male haploloads of the sons is assumed to be highest. That, however, does not exclude a certain degree of mother-daughter microchimerism at the same time which may even be theoretically expected by powerful mother-son microchimerism^49,50^. Most likely so in Pathway 3, where the numbers of affected males and females in PA and MA are as expected. No birth order effect, nearly no affected parent-offspring pairs, and no sib concordance were seen in the Faroese material, where the HLA compatibility between mother-offspring and father is assumed to be relatively high due to consanguinity so that the microchimerism reaction is relatively weak. In consanguinity, the difference between maternal and paternal haploloads of the fetus may furthermore be evened out because both parents theoretically may confer different imprinted susceptibility, causing alterations in the stochastic, independent regulation of the proband^49^.

The statistical power of our findings can be discussed, especially since the patients have been sorted up in many diagnostic subsets and transgenerational groups. Compared with thousands of investigated patients with MBD from cancer registries and from genome-wide association studies, our patients represent small sample sizes. However, our findings get strength from being systematically cross-checked over several generations. This is especially true of the Faroese material, as far as we know the largest consanguineous population with MBD described up to now.

It is not really surprising to see parental genomic imprinting and microchimeristic genomic changes as the predicted mechanisms of the model, because both are well known to be related to MBD.

Parental genomic imprinting is seen in the segregation of mammalian growth factor genes^50,51^, where subsets of genes required for the development are expressed at specific stages of life and in specific tissues from only one chromosome copy, depending upon the parental origin of that chromosome copy^49–52^. Parental genomic imprinting has earlier been discussed as a possible mechanism behind the non-Mendelian genomic output in CLL^53^. A common feature in the segregation of this group of genomic imprinted genes is a pronounced different outcome in paternal and in maternal imprinting. One classic example is the Prader-Willi and Angelman syndromes^54^, caused by a partial deletion of chromosome 15 in most of the cases. If a fetus receives the chromosome with the deletion from the father, and the inactivated, imprinted copy of the gene from the mother (maternal imprinting), the fetus will have Prader-Willi syndrome. In contrast, a fetus receiving the partially deleted chromosome from the mother and the imprinted gene from the father (paternal imprinting) will have Angelman syndrome. Similarly, MBD is a syndrome closely related to the growth of monoclonal hematopoietic cells. Women with CLL^45,46^ or NHL^55^ have in general a better prognosis than male patients, including a better response to treatment with less toxicity and generally a higher age at onset of disease. These differences in MBD between the sexes, otherwise not definitely explained, could be seen as a different inheritance of susceptibility according to pathway 1 to 3. All known systems of meiotic drive (Non-Mendelian segregation) cause segregation ratios that are different between the sexes^49–52^.

The relationship between alterations in growth factors and MBD is also seen, most likely, from the combined data on the high frequency of MBD on the Faroe Islands, the high birthweight and the high rate of miscarriages and stillborn (Cf. Material and Methods, “the Faroe Islands, Families and Sampling History”)^29–31^. A relationship between development of MBD (ALL is the most studied) and a high body weight of the patient as newborn has been shown in other studies as well as an increased frequency of miscarriages and abortions in mothers to MBD patients^56,57^.

In such systems of interacting genes, modifier genes may invade the system during the evolution^50,52,58,59^. Therefore, a modifier to our model was theoretically expected. Microchimerism was an option in our pattern recognition for such a modifier. Microchimerism has previously been described in MBD as a mechanism for development of autoimmunity^40–44^, graft-versus-leukemia effect^60^, and the mechanism of birth order effect due to increased acquired tolerance of the mother in step with her parities and the number of her male partners^40,61,62^. Also, the specific type of microchimerism in MBD has been discussed in the light of tolerance related to the degree of tissue-type compatibility (HLA-match) between mother, offspring and father^60,62^. Microchimerism has furthermore been related to the pathophysiology of some types of solid cancer, e.g. breast cancer^63^.

Microchimeristic susceptibility could be one mechanism behind anticipation, seen in the accumulation of susceptibility in the pedigree with increased genomic diversity in “the genomic landscape” down through the generations. Anticipation can be interpreted as a process that enhances the expression of the susceptibility. It is fascinating that the model also suggests a strategy for loss of susceptibility and thereby a delicate balance between genomic accumulation and depletion, so that affected families after a number of generations will not be overloaded with susceptibility. Anticipation produces an increased number of high-malignant diseases, especially in younger patients. ALL, having a high frequency in the inbred Faroes material (Table 1), is such an example with a very high mortality if untreated. In such cases, death leads to depletion of susceptibility in the population before and during fertile life.

The familial clustering of HL, seen in the present study (cf. Results, co- and contravariations) has previously been reported^18^ and ascribed to familial HLA-association^12^. Also, the modest risk of familial NHL seen in the present study (percentage of familial FL and DLBCL below the percentage of FL and DLBCL reported by the Cancer Registries, Table 1) has been described^7^. Comparing the findings in the Faroese family (related parents) with the families with MBD in Norway and Denmark (unrelated parents), HL and DLBCL have the highest percentage in the Faroese family where both parents due to inbreeding theoretically can transfer interfering susceptibility^52^.

In conclusion, the model suggested is an operational interaction between parental genomic imprinting of susceptibility genes and mother-son microchimerism. Theoretically, the intensity of the mother-son microchimerism depends on the tissue-type compatibility among mother, sons and the father. This may well explain why the segregation has been observed to be tardive with related parents, and immediate (from parent to offspring) with unrelated parents. The model explains the male predominance, the unique pathway 2 in the segregation of susceptibility to chronic lymphocytic leukemia, and draws attention to the relevance of growth factor genes for monoclonal expansion.

## Data Availability

Data are stored at the National Norwegian Cancer Registry, Ullernchausseen 64, NO 0379, Oslo, Norway, att. Dr. Tom Børge Johannesen.
